# Aerobic Exercise Attenuated Bleomycin-Induced Lung Fibrosis in Th2-Dominant Mice

**DOI:** 10.1371/journal.pone.0163420

**Published:** 2016-09-27

**Authors:** Adilson Santos Andrade-Sousa, Paulo Rogério Pereira, BreAnne MacKenzie, Manoel Carneiro Oliveira-Junior, Erasmo Assumpção-Neto, Maysa Alves Rodrigues Brandão-Rangel, Nilsa Regina Damaceno-Rodrigues, Elia Garcia Caldini, Ana Paula Pereira Velosa, Walcy Rosolia Teodoro, Ana Paula Ligeiro de Oliveira, Marisa Dolhnikoff, Oliver Eickelberg, Rodolfo Paula Vieira

**Affiliations:** 1 Laboratory of Pulmonary and Exercise Immunology (LABPEI) and Brazilian Institute of Teaching and Research in Pulmonary and Exercise Immunology (IBEPIPE), Nove de Julho University (UNINOVE), Rua Vergueiro, 235/249, São Paulo – SP, Brazil; 2 Laboratory of Cellular Biology (LIM 59), School of Medicine, University of São Paulo, Avenida Doutor Arnaldo, 455, Sao Paulo – SP, Brazil; 3 Laboratory of Medical Investigation (LIM 17), School of Medicine, University of São Paulo, Avenida Doutor Arnaldo, 455, Sao Paulo – SP, Brazil; 4 Department of Pathology, School of Medicine, University of São Paulo, Avenida Doutor Arnaldo, 455, Sao Paulo – SP, Brazil; 5 Comprehensive Pneumology Center (CPC), Ludwig Maximilian Universität München and Helmholtz Zentrum München, Max-Lebsche-Platz 31, München, Germany; Forschungszentrum Borstel Leibniz-Zentrum fur Medizin und Biowissenschaften, GERMANY

## Abstract

**Introduction:**

The aim of this study was to investigate the effect of aerobic exercise (AE) in reducing bleomycin-induced fibrosis in mice of a Th2-dominant immune background (BALB/c).

**Methods:**

BALB/c mice were distributed into: sedentary, control (CON), Exercise-only (EX), sedentary, bleomycin-treated (BLEO) and bleomycin-treated+exercised (BLEO+EX); (n = 8/group). Following treadmill adaptation, 15 days following a single, oro-tracheal administration of bleomycin (1.5U/kg), AE was performed 5 days/week, 60min/day for 4 weeks at moderate intensity (60% of maximum velocity reached during a physical test) and assessed for pulmonary inflammation and remodeling, and cytokine levels in bronchoalveolar lavage (BAL).

**Results:**

At 45 days post injury, compared to BLEO, BLEO+EX demonstrated reduced collagen deposition in the airways (p<0.001) and also in the lung parenchyma (p<0.001). In BAL, a decreased number of total leukocytes (p<0.01), eosinophils (p<0.001), lymphocytes (p<0.01), macrophages (p<0.01), and neutrophils (p<0.01), as well as reduced pro-inflammatory cytokines (CXCL-1; p<0.01), (IL-1β; p<0.001), (IL-5; p<0.01), (IL-6; p<0.001), (IL-13; p<0.01) and pro-fibrotic growth factor IGF-1 (p<0.001) were observed. Anti-inflammatory cytokine IL-10 was increased (p<0.001).

**Conclusion:**

AE attenuated bleomycin-induced collagen deposition, inflammation and cytokines accumulation in the lungs of mice with a predominately Th2-background suggesting that therapeutic AE (15–44 days post injury) attenuates the pro-inflammatory, Th2 immune response and fibrosis in the bleomycin model.

## Introduction

Idiopathic pulmonary fibrosis (IPF) affects primarily men in the 5^th^ decade of life at a rate of 4-12/100,000, and has a prognosis of 3–5 years following diagnoses [1]. Patients present dyspnea due to expanding fibrotic lesions caused by the accumulation of extracellular matrix proteins in the lung parenchyma, which gradually destroys alveoli leading to insufficient gas exchange. While surfactant protein folding defects are responsible for a small percentage of pulmonary fibrosis [2], most cases are idiopathic. Studies suggest that repetitive epithelial injury caused by environmental or endoplasmic reticulum (ER) stress, combined with an aberrant wound repair mechanism may be partly to blame but the exact mechanisms remain unknown [3,4]. While some drugs slightly reduce the rate of lung function decline, treatment options remain palliative [5]. Though the role of inflammation in IPF, specially from Th2 background, including the treatment of IPF with anti-inflammatories is hotly debated [6], the expression of Th2 cytokines, specifically IL-4 and IL-5, predominate over Th1 cytokine, IFN-gamma, as accredited to play an important role for progression of pulmonary fibrosis [7].

Aerobic exercise (AE) has been found to reduce Th2-mediated inflammation in murine allergic asthma models [8,9] and some clinical studies with allergic asthma patients show that exercise may be beneficial [10–12]. In the context of IPF, recent clinical studies have highlighted that while exercise does not cure IPF, pulmonary rehabilitation programs that incorporate physical training improve the patient’s six-minute walk scores, body composition, and quality of life [13–15] with some patients responding more positively than others.

Though bleomycin was originally used as an anti-cancer drug, given its DNA cleaving and anti-tumorigenic activity, it’s systemic use was repealed due to the occurrence of a lethal, bleomycin-induced pulmonary fibrosis side effect [16,17]. Oro-tracheal administration of bleomycin in rodents is currently the most utilized animal model of IPF as the lesions formed due to bleomycin are histologically similar to those observed in IPF [18]. However, the model is limited, as mice are able to repair the lesions over time. The rate of repair however is often used to indicate whether a particular molecule, pathway or treatment could potentially be beneficial to IPF patients. While two studies have shown that chronic, moderate AE attenuates bleomycin-induced fibrosis [19,20], this study uses mice of a Th2-dominant immune-responsive background (BALB/c) to test for the first time, the hypothesis that AE accelerates the resolution of bleomycin-induced airway fibrosis in part by attenuating the Th2 immune response.

## Methods

We also state that all experiments were approved by the ethical committees of the University of Sao Paulo School of Medicine and Nove de Julho University (375/13). Experiments were carried out in accordance to the Guide for the Care and Use of Laboratory Animals, published by the U.S. National Institutes of Health (NIH publication no. 85–23, revised 1996). During the whole experiment, the animals did not present any alteration in health status, which was monitored 5x/week prior the physical training sessions. Also, no mice died during the experiments.

### Animals and experimental groups

BALB/c, male mice (20–25 g) were obtained from the Central Animal Facility of School of Medicine of the University of Sao Paulo and distributed into Control (Con), Exercise (Ex), Bleomycin (Bleo) and Bleomycin+Exercise (Bleo+Ex) groups (n = 8/group).

### Bleomycin-induced pulmonary fibrosis protocol

Bleomycin sulfate (1.5UI/kg; Meizler Biopharma, SP, Brazil) was administered oro-tracheally under anesthesia (ketamine 100mg/kg and xylazine 10mg/kg).

### Exercise test and aerobic training protocol

The aerobic training protocol began 15 days after bleomycin administration. Adaptation to treadmill training was performed as previously described [8,21]. Following 3 days of adaptation (15 min/day, 25° incline, 0.2 Km/h), animals were submitted to a physical test (beginning at 0.2 Km/h, increasing 0.1 Km/h every 2.5 minutes) until animals were exhausted. Exhaustion was defined as failure to run following 10 gentle, mechanical stimuli [8,22]. Treadmill training was performed at 60% of the maximum velocity reached in the physical test during 4 weeks, 5x/week, 60 minutes/session. Twenty-four hours before euthanasia (ketamine 200mg/kg and xylazine 20mg/kg), the final physical test was performed [8,22].

### Quantification of collagen fibers in the airways wall and in the lung parenchyma

Following transcardiac de-sanguination with phosphate buffered saline (PBS), left lobe were excised, fixed in 4% formalin solution, embedded in paraffin and sectioned in five micrometers slices. The serial sections were done in approximately 2mm deep into the lung tissue to assure that central to distal airways would be reached in the sections. Picro Sirius Red staining for collagen fibers was performed as previously described [9,22–24]. Collagen fibers were detectable via light microscopy (red staining) and the extent of collagen content was determined in the walls of five airways per mouse, in all 8 mice of each group and also in fifteen lung parenchyma fields per mouse (at 20X magnification) using the Image Pro Plus 4.5 software.

First, to assess collagen content within the airway wall, the airway area (defined as the area between the epithelial basement membrane and airway adventitia) was delimited; vessels were excluded. Next, a constant color threshold was calculated for the analyses of all samples by subtracting the difference of staining intensity between controls and fibrotic animals. This threshold was calculated by the software and was used consistently to measure the areas of red staining. Airway collagen content was calculated as the relation between the area of red staining within the airway wall and the total airway area [9,22–24] and results expressed as percentage. Airway collagen content in each animal was calculated as mean values of 5 airways.

For quantification of collagen content in the lung parenchyma, first the total area of lung parenchyma in each field was determined and also the area of air spaces. The parenchymal tissue area was determined in each field by subtracting the air spaces area from the total parenchymal area. Then, using a constant color threshold, the red staining area was determined within the lung parenchyma (15 fields per animal). Parenchyma collagen content in each field was then calculated as the relation between the parenchymal area of collagen fibers and the total parenchyma area in the field and expressed as percentage.

### Collection and analyses of bronchial alveolar lavage (BAL) fluid

Following anesthetization, a cannula was inserted into the trachea and lungs were washed with 2x 500ml of PBS. BAL fluid was centrifuged at 900g for 10 minutes at 4°C. Supernatant was stored at -80°C for further ELISA experiments, and the cell pellet was resuspended in 1ml of PBS for total cell count (Neubauer chamber) and differential cell count analyses (cytospin preparation). Cytospins were stained with Diff Quick and differential cell counts were performed based on hematological criteria, considering 300 cells [25].

### Cytokines Measurements in BAL Fluid

The levels ofIL-1β (432603; Biolegend, CA, USA), IL-5 (431203; Biolegend, CA, USA), IL-6 (431303; Biolegend, CA, USA), CXCL-1/KC (DY453; RD Systems, MN USA), IL-10 (431413; Biolegend, CA, USA), IL-13 (DY413; RD Systems, MN, USA) and IGF-1 (DY791; RD Systems, MN USA), were measured in BAL fluid using ELISA kits according to the manufactures’ recommendations.

### Statistical analysis

The software Graph Pad Prism 5.0 was used to perform statistical analysis and also for graphs. The normality of the data was tested by Shapiro-Wilk test and the data was analyzed by one-way analysis of variance (ANOVA) followed by the Bonferroni post-hoc test. Significance levels were considered for p<0.05. Values were expressed as mean ± SD.

## Results

### Collagen deposition in the airways and in the lung parenchyma was reduced by aerobic exercise

**Fig 1:**
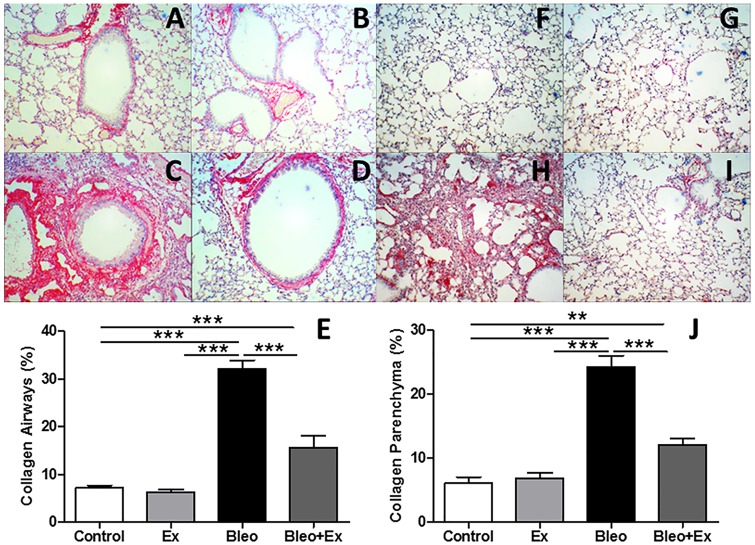
Collagen deposition in the airways and in the lung parenchyma. (A–D) Representative light microscopy images (20X) of Picro Sirius Red staining (red indicates collagen expression) in the airways wall of: Control, Ex, Bleo, Bleo+Ex groups, respectively) and in the (F–I) lung parenchyma; Control, Ex, Bleo, Bleo+Ex groups, respectively). (F) Quantitative analysis of collagen depositon in the airways and (J) in the lung parenchyma. These observations (n = 8/group) were quantified by image analysis software. ***p<0.001 and **p<0.01. Scale bars: 100um.

Picro Sirius Red staining (red indicates collagen expression) was used to visualize the extent of collagen deposition in the airways wall in sedentary (CON) animals (Fig 1A), exercise only (EX) (Fig 1B), sedentary bleomycin (BLEO) (Fig 1C), and exercised bleomycin animals (BLEO+EX) (Fig 1D). An increase in collagen fibers was observed in BLEO mice compared to CON and EX while BLEO+EX mice showed less collagen than the BLEO group. In summary, these observations (n = 8/group) were quantified by image analysis software (Fig 1E) and showed that aerobic exercise (AE) decreased the extent of bleomycin-induced collagen deposition. In addition, parenchymal remodeling was also evaluated in sedentary (CON) animals (Fig 1F), exercise only (EX) (Fig 1G), sedentary bleomycin (BLEO) (Fig 1H), and exercised bleomycin animals (BLEO+EX) (Fig 1I). An increase in collagen fibers in the lung parenchyma was observed in BLEO mice compared to CON and EX while BLEO+EX mice showed less collagen than the BLEO group. In summary, these observations (n = 8/group) were quantified by image analysis software (Fig 1J) and showed that aerobic exercise (AE) decreased the extent of bleomycin-induced collagen deposition.

### Aerobic exercise decreased bleomycin-induced inflammatory cells in BAL

**Fig 2:**
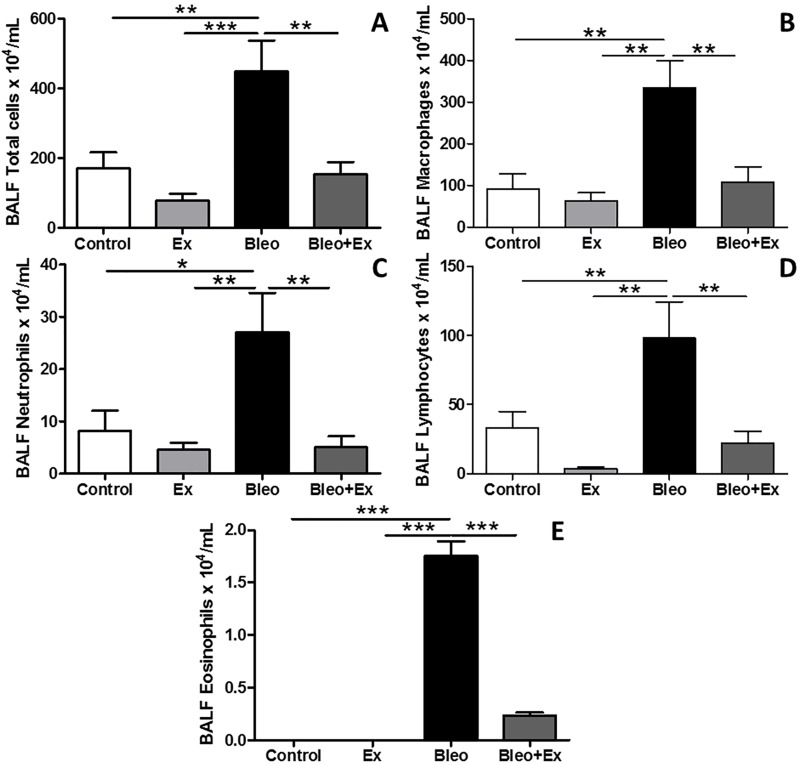
Total and differential cell counts of inflammatory cells in BAL. Total and differential analyses of immune cells (n = 8/group) in bronchoalveolar lavage (BAL) fluid in Sedentary (CON), exercise-only (EX), sedentary bleomycin (BLEO), and exercised bleomycin animals (BLEO+EX). (A) Total cell count, (B) macrophages, (C) neutrophils, (D) lymphocytes, and (E) eosinophils. ***p<0.001; **p<0.01 and *p<0.05.

In Fig 2, total and differential cell counts of bronchoalveolar lavage (BAL) fluid (n = 8 animals/group) were analyzed. Total cell count was slightly reduced in EX group compared to CON animals (Fig 2A) but not to a statistically significant extent. An increase in the total number of cells in the BAL fluid of BLEO mice was detected (Fig 2A). In BLEO+EX, this number was reduced to the levels of the CON group (Fig 2A). Differential cell counts for macrophages (Fig 2B), neutrophils (Fig 2C) and lymphocytes (Fig 2D) showed the same pattern: compared to CON, EXE only mice showed a trend towards a decrease in immune cells, while cell counts were significantly augmented in BLEO only mice and reduced in BLEO+EX compared to BLEO only. Very few eosinophils were detected in CON and EX mice compared to BLEO mice (Fig 2B). Eosinophil number was decreased in BLEO+EX mice compared to BLEO mice (Fig 2E). In summary, AE reduced the number of bleomycin-induced inflammatory cells in BAL.

### Aerobic exercise diminished the expression of bleomycin-induced pro-inflammatory cytokines and IGF-1 in BAL

**Fig 3:**
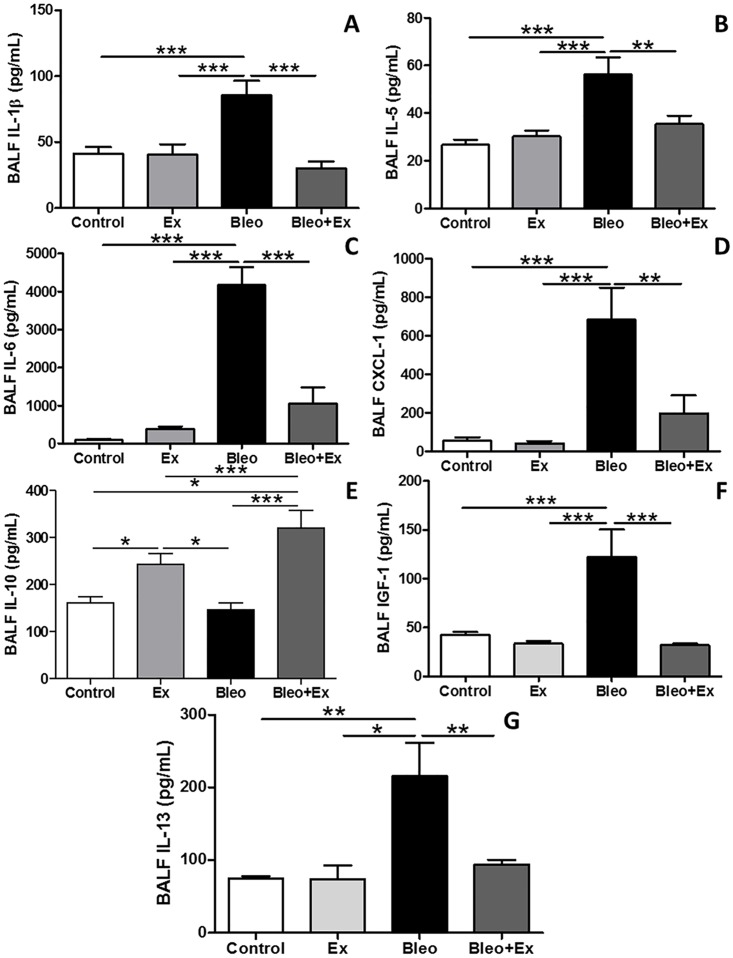
Cytokine and growth factor levels in BAL. Cytokines and a growth factor in BAL were analyzed by ELISA (n = 8/group); Sedentary (CON), exercise-only (EX), sedentary bleomycin (BLEO), and exercised bleomycin animals (BLEO+EX). (A) IL-1β, (B) IL-5, (C) IL-6, (D) CXCL-1, (E) IL-10, (F) IGF-1 and (G) IL-13. ***p<0.001; **p<0.01 and *p<0.05.

The pro-inflammatory cytokine IL-1β, was increased in BLEO and decreased in BLEO+EX (Fig 3A). Pro-inflammatory cytokines IL-5, IL-6, and IL-13 showed were also increased in BLEO and decreased in BLEO+EX (Fig 3B, 3C and 3G respectively). The neutrophil chemo-attractant CXCL-1/KC was increased in BLEO mice and reduced in BLEO+EX group (Fig 3D). Anti-inflammatory cytokine IL-10 was increased in EX compared to CON and further increased in BLEO+EX compared to all groups (Fig 3E). Insulin-like growth factor 1 (IGF-1), implicated as a potent, pro-fibrotic, fibroblast growth factor in the context of IPF and bleomycin-induced fibrosis, was increased in the BLEO group, and reduced in the BLEO+EX group (Fig 3F).

## Discussion

Using a Th2-dominant immune-responsive background (BALB/c), this study tested for the first time the hypothesis that AE accelerates the resolution of bleomycin-induced airway fibrosis *in part* by attenuating the Th2 response, as demonstrated by reduced levels of IL-5 and IL-13. While other strains of mice also mount a mild Th2 inflammatory cytokine response to bleomycin treatment, the Th2 reaction is significantly stronger in BALB/c mice than in other strains [26–28]. Though the role of Th2 cytokines in IPF is debated; as patients present with very little overt inflammation at the time of diagnosis, inflammation is suspected to play a role in the early stages of disease [23]. Though mere overexpression of IL-13 in mice lead to increased fibrosis [24], and a clinical study using anti-IL-13 therapy is currently underway (NCT01872689), it is important to note that so far, therapies attenuating either Th1 [29] or Th2 inflammation [30,31] do not generally lead to better overall outcomes for IPF patients [32]. Therefore, if increased Th2 inflammation indeed contributes to the onset of IPF, exercise-induced attenuation of Th2 inflammation may be more beneficial as a preventative therapy. However, the anti-fibrotic effects of exercise are not limited to the immune system as demonstrated in a recent study which implicated decreased 5-serotonin and Akt-signaling in the reduction of fibrosis [33]. In summary, further studies are needed in order to fully understand the extent and mechanisms by which aerobic exercise attenuates fibrosis.

In a more recent study, C57Bl/6 (Th1-dominant background) mice were subjected to the same treadmill protocol used in the present study (moderate intensity running at 60% maximal capacity, for 4 weeks, beginning at 15 days post-injury) [20]. C57Bl/6 (BLEO+EX) also showed decreased inflammation and fibrosis, and attenuated Akt and serotonin signaling compared to BLEO. Both Akt and serotonin pathways promote and sustain fibroblast growth. While a reduction in collagen fibers in the lung parenchyma (p<0.01) was reported in the C57Bl/6 study, they were reduced to a lesser extent than in this study (p<0.001). However, the accuracy of collagen content measurement reported by this study is limited due to the low proportion of actual 2D areas measured versus total lung area and the lack of stereological methods [34].

In addition, the study of Pereira et. al., also observed that AE reduced the influx of inflammatory cells, except for eosinophils, a classical inflammatory cell involved in allergic inflammation. Furthermore, pro-inflammatory cytokines: CXCL1/KC, IL-1beta, and IL-6 were reduced. However, the reduction in inflammation also occurred to a lesser extent than in BALB/c mice despite the use of the same treatment protocols. Pereira et. al., observed an increase in anti-inflammatory cytokine IL-10 due to AE as well, but also to a lesser extent than in BALB/c mice used in this study. These differences highlight not only the potent anti-inflammatory effect of AE in a Th2 dominant background vs. a Th1 dominant background but also the significant affect that heterogeneous immune compositions may have on IPF patient treatment responses to pulmonary rehabilitation therapy. As observed in recent, preliminary clinical studies with exercise and IPF patients, some individuals respond more positively than others to exercise [13,35]. Therefore, it may be prudent for future clinical studies that combine IPF and therapeutic exercise to examine the levels of Th2 cytokines in blood or BAL.

A precedent has already been set in the literature for the ability of exercise to dampen Th2-mediated immune responses, especially in the context of allergic asthma. For example, in ovalbumin-treated mice, just a single bout of exercise decreased ovalbumin-induced Th2 cytokines IL-5 and IL-13 [36]. Extended protocols have shown a reduction in eosinophils, CD3+ and CD4+ lymphocytes, CXCL-1, IL-4, IL-5, IL-6, IL-13, resulting in a decrease in airway remodelling, mucus synthesis, smooth muscle thickness, and airway resistance [25,37,38]. Therefore, not surprisingly, in the present study, pro-inflammatory IL-13 and IL-5, the neutrophil chemo-attractant CXCL-1, as well as the total number of eosinophils, lymphocytes and neutrophils, were all reduced in BLEO+EX compared to BLEO alone.

While eosinophilia and IL-4 expression may be expendable in rodent fibrosis models [39] IL-4 itself is neither particularly elevated in sera [40], nor in BAL of end-stage IPF patients [41]. However, receptors IL-4Rα, as well as IL-13 receptors: IL-13Rα2, and IL-13Rα1, are upregulated in fibroblastic foci of IPF patients [42]. IL-13, is a pro-fibrotic, Th2 cytokine that increases collagen synthesis in fibroblasts and its expression may correlate with the severity of IPF [43,44]. *IL-13*^-/-^ mice are in fact protected from FITC-induced fibrosis [45]. Clinical studies using Tralokinumab, a human recombinant monoclonal antibody against IL-13 are in progress [46,47]. Therefore, attenuation of IL-13 by moderate AE may be an important therapeutic benefit of pulmonary rehabilitation for IPF patients.

This study also looked for the first time at the expression of insulin-like growth factor 1 (IGF-1), a pro-fibrotic growth factor, in the context of AE and bleomycin injury. Low intensity exercise has been shown to decrease IGF-1 levels in low-intensity exercisers after a period of six-weeks and is associated with decreased risk of cardiovascular disease [48]. A small trend towards a reduction was observed in EX compared to CON and a significant reduction was observed in BLEO+EX compared to BLEO. In non-IPF patients, IGF-1 localized exclusively to alveolar macrophages. In contrast, in IPF-patients, the domain of IGF-I expression is expanded to interstitial macrophages, alveolar epithelial cells, and ciliated columnar epithelial cells. IGF-1 expression by interstitial macrophages was found to correspond positively to the level of fibrosis in IPF patients [49]. In this model, AE not only reduced IGF-1 but also IL-4 and IL-13 which have been shown to stimulate IGF-1 in macrophages and myofibroblasts [50]. Furthermore, AE also resulted in a decrease in the number of macrophages in BLEO+EX lungs thus reducing a potential source of pro-fibrotic IGF-1.

In addition, this study also evaluated pro-inflammatory cytokine IL-1β expression as genetic over-expression models can cause a pulmonary fibrotic phenotype [51] similar to bleomycin-induced fibrosis. In both IL-1β and bleomycin-induced fibrosis in rodents, the development of fibrosis was IL-17 dependent [52]. Both IL-17 and IL-1β expression are increased in IPF patient BAL [53]. Interestingly, Wilson et. al., also demonstrated that IL-10 inhibits the pro-inflammatory, pro-fibrotic IL-23–IL-17A pathway rather than the IL-12–IFN-γ (Th1) axis. Although IL-17 was not measured in the current study, taken together, the increase in IL-10 in the BLEO+EX group likely had a strong anti-inflammatory and anti-fibrotic effect due to inhibition of IL-1β by IL-10. AE-dependent increases in IL-10 may also be responsible for the reduction of IL-6 in the BLEO+EX group as IL-6 is known to induce IL-10, and IL-10 in turn inhibits IL-6. While there was significant variability found in the expression of IL-1β and IL-6 in alveolar macrophages isolated from IPF patients [54] increased levels of these cytokines in BAL fluid are associated with IPF [53,55]. Interestingly, IPF patients with polymorphisms in IL-10 which may affect the efficiency of IL-10 translocation and signal peptide cleavage have been identified [56] which may contributing to a pro-inflammatory environment. Thus, in IPF patients with these IL-10 polymorphisms, it would be unlikely that an AE-induced increase in IL-10 alone could attenuate an aberrant Th2 immune response. Furthermore, though genetic IL-10 overexpression was found to attenuate inflammation and fibrosis in mice [57,58], lung-specific overexpression of IL-10 was found to cause fibrosis in a STAT-independent manner [59]. Therefore, a tight regulation of Th2 cytokines is likely important for fibrosis repair.

## Conclusions

These results support the significance of the individual immune response in the context of a complicated, heterogeneous pulmonary disease: IPF. These findings suggest that future IPF and exercise studies should more closely examine not only the Th2 cytokine levels in patients who respond to exercise, but also additional potential mechanisms by which exercise may have a more positive outcome in particular individuals. Finally, although increased inflammation in IPF surgical lung biopsies predict poorer disease outcome [60] and many studies suggest that inflammation may play a role in acute exacerbations of IPF [61], an occurrence which often ends in death [31], corticosteroids are not an effective treatment for IPF patients [30]. Therefore, although moderate exercise decreased the Th2 immune response in this model, whether or not inhibition of Th2 cytokines was the most important exercise-induced anti-fibrotic effect remains unknown. Future studies should investigate alternative, beneficial exercise-induced modulations in growth factors pathways, hormones, apoptosis, and cell survival pathways, which may have a central role in exercise-mediated anti-fibrotic affects.

## Supporting Information

S1 File Raw data is available for Figs 1A–1H, 2A–2E and 3A–3G. (DOCX)
