# Supplementary material for: Aerobic Exercise Attenuated Bleomycin-Induced Lung Fibrosis in Th2-Dominant Mice
Source: PLoS One. 2016 Sep 27;11(9):e0163420. doi: 10.1371/journal.pone.0163420 (PMC5038953; doi:10.1371/journal.pone.0163420)
Supplement: S1 File — (DOCX) [file pone.0163420.s001.docx]

**São Paulo, 07 September 2016.**

**To PLOS One**

**STATEMENT ABOUT DATA AVAILABILITY**

We state that all data obtained from the experiments performed that resulted in the manuscript entitled “Aerobic Exercise Attenuated Bleomycin-Induced Lung Fibrosis in Th2-dominant Mice” are fully available bellow and also upon request, if any details is needed.

On behalf of all co-authors,

Sincerely,

**Rodolfo de Paula Vieira, Prof. Dr**

**Laboratory of Pulmonary and Exercise Immunology**

**Nove de Julho University - São Paulo – SP – Brazil**

**Data regarding**:

**Fig 1A-D Airway collagen**

| Control | Ex | Bleo | Bleo+Ex |
| --- | --- | --- | --- |
| 8,58740 | 8,68554 | 25,1147 | 12,3578 |
| 7,35490 | 6,14780 | 29,1445 | 15,4569 |
| 6,27554 | 5,89970 | 31,4796 | 8,1112 |
| 5,15280 | 4,11450 | 36,2235 | 22,4669 |
| 7,24780 | 6,87550 | 33,4766 | 26,4778 |
| 8,12480 | 7,11450 | 39,1145 | 17,1148 |
| 9,47890 | 5,94550 | 32,1236 | 9,1023 |

**Fig 1E-H Parenchymal collagen**

| Control | Ex | Bleo | Bleo+Ex |
| --- | --- | --- | --- |
| 8,8896 | 8,9865 | 25,6544 | 9,8745 |
| 8,7445 | 7,8854 | 31,4447 | 12,4568 |
| 6,2254 | 9,5564 | 17,8895 | 15,4633 |
| 7,5688 | 4,5663 | 24,1154 | 11,4540 |
| 3,4457 | 7,4569 | 19,66654 | 10,4789 |
| 4,8896 | 5,6321 | 23,4587 | 15,4442 |
| 2,7789 | 4,1557 | 27,6654 | 9,6654 |

**Fig 2A Total cells in BAL**

| Control | Ex | Bleo | Bleo+Ex |
| --- | --- | --- | --- |
| 300, | 50, | 150, | 170, |
| 130, | 110, | 650, | 130, |
| 90, | 180, | 450, | 70, |
| 50, | 60, | 70, | 340, |
| 260, | 60, | 300, | 70, |
| 320, | 50, | 750, | 180, |
| 50, | 40, | 660, | 120, |
|  |  | 560, |  |

**Fig 2B Macrophages in BAL**

| Control | Ex | Bleo | Bleo+Ex |
| --- | --- | --- | --- |
| 234,0 | 45,0 | 136,5 | 74,8 |
| 22,1 | 107,8 | 500,5 | 114,4 |
| 16,2 | 154,8 | 261,0 | 16,1 |
| 12,0 | 55,8 | 52,5 | 306,0 |
| 101,4 | 56,4 | 279,0 | 56,0 |
| 217,6 | 0,0 | 585,0 | 88,2 |
| 38,5 | 25,6 | 488,4 | 105,6 |
|  |  | 369,6 |  |

**Fig 2C Neutrophils in BAL**

| Control | Ex | Bleo | Bleo+Ex |
| --- | --- | --- | --- |
| *27,0** | 1,5 | 22,1 | 2,50 |
| 0,0 | 0,0 | 15,2 | 1,10 |
| 0,0 | 8,1 | 18,4 | 18,00 |
| 0,5 | 9,8 | *10,2** | 1,20 |
| 20,8 | 6,0 | 22,1 | 1,80 |
| 22,4 | 7,5 | 72,0 | 0,00 |
| 2,0 | 3,3 | 16,0 | 6,00 |
| 12,0 | 0,0 | 23,5 | 9,55 |

**Fig 2D Lymphocytes in BAL**

| Control | Ex | Bleo | Bleo+Ex |
| --- | --- | --- | --- |
| 39,0 | 4,5 | 12,0 | 73,1 |
| 70,2 | 1,1 | 149,5 | 10,4 |
| 4,5 | 7,2 | 112,5 | 6,3 |
| 3,5 | 3,0 | 7,7 | 23,8 |
| 26,0 | 1,8 | 15,0 | 11,9 |
| 80,0 | 0,0 | 157,5 | 19,8 |
| 9,5 | 8,8 | 138,6 | 8,4 |
|  |  | 190,4 |  |

**Fig 2E Eosinophils in BAL**

| Control | Ex | Bleo | Bleo+Ex |
| --- | --- | --- | --- |
| 0, | 0, | 1,895 | 0,354 |
| 0, | 0, | 2,115 | 0,270 |
| 0, | 0, | 2,210 | 0,195 |
| 0, | 0, | 1,915 | 0,190 |
| 0, | 0, | 1,987 | 0,175 |
| 0, | 0, | 1,120 | 0,285 |
| 0, | 0, | 1,470 | 0,290 |
| 0, | 0, | 1,310 | 0,115 |

**Fig 3A IL-1beta in BAL**

| Control | Ex | Bleo | Bleo+Ex |
| --- | --- | --- | --- |
| 52,340 | 37,373 | 65,518 | 8,337 |
| 35,857 | 88,905 | 75,828 | 42,242 |
| 28,709 | 35,449 | 70,954 | 38,493 |
| 38,352 | 31,483 | 45,237 | 44,027 |
| 53,161 | 47,861 | 110,670 | 15,454 |
| 56,849 | 23,725 | 100,134 | 24,643 |
| 23,498 | 46,155 | 129,586 | 36,543 |
|  | 12,253 |  |  |

**Fig 3B IL-5 in BAL**

| Control | Ex | Bleo | Bleo+Ex |
| --- | --- | --- | --- |
| 38,570 | 20,914 | 41,700 | 33,445 |
| 28,393 | 37,297 | 77,119 | 30,877 |
| 25,005 | 35,741 | 41,029 | 49,167 |
| 24,504 | 26,152 | 40,862 | 47,868 |
| 25,384 | 28,528 | 59,135 | 23,996 |
| 21,241 | 37,613 | *36,514** | 31,588 |
| 23,656 | 25,431 | 77,376 | 31,042 |

**Fig 3C IL-6 in BAL**

| Control | Ex | Bleo | Bleo+Ex |
| --- | --- | --- | --- |
| 21,84215 | 472,5670 | 5484,06 | 695,0938 |
| 35,79885 | 410,4290 | 3118,708 | 3215,0100 |
| 104,753 | 156,9380 | 3385,69 | 115,0830 |
| 207,701 | 423,1260 | 3518,14 | 705,1190 |
| 192,8991 | 653,7960 | 4647,73 | 1838,7000 |
| 141,540 | 163,6100 | 3204,183 | 102,8187 |
| 3,5008 | 422,0806 | 6637,96 | 728,3700 |
|  |  | 3383,16 |  |

**Fig 3D CXCL-1 in BAL**

| Control | Ex | Bleo | Bleo+Ex |
| --- | --- | --- | --- |
| 31,331930 | 17,242190 | 1584,041000 | 36,087380 |
| 29,899290 | 52,694250 | 515,801700 | 52,961670 |
| 178,834200 | 26,450530 | 960,509300 | 109,806000 |
| 59,253360 | 91,716240 | 360,873800 | 199,393500 |
| 28,536370 | 96,979480 | 152,694200 | 74,964440 |
| 36,019290 | 17,916270 | 1013,638000 | 204,937000 |
| 61,141470 | 24,645260 | 324,409200 | 78,544440 |
| 27,001170 | 18,583100 | 554,966400 | 830,477400 |

**Fig 3E IL-10 in BAL**

| Control | Ex | Bleo | Bleo+Ex |
| --- | --- | --- | --- |
| 241,444 | 358,065 | 220,146 | 296,530 |
| 164,848 | 227,307 | 115,704 | 209,099 |
| 124,013 | 216,522 | 113,271 | 233,386 |
| 142,022 | 138,853 | 138,853 | 226,103 |
| 172,893 | 270,861 | 114,079 | 537,576 |
| 137,556 | 238,946 | 201,748 | 375,458 |
| 137,507 | 225,712 | 100,865 | 321,456 |
| 169,346 | 272,095 | 158,725 | 361,244 |

**Fig 3F IGF-1 in BAL**

| Control | Ex | Bleo | Bleo+Ex |
| --- | --- | --- | --- |
| 55,692 | 34,480 | 69,281 | 30,859 |
| 44,497 | 21,828 | 48,327 | 33,253 |
| 39,116 | 40,964 | 114,824 | 28,301 |
| 35,214 | 38,871 | 269,144 | 35,008 |
| 53,114 | 31,405 | 120,211 | 34,904 |
| 41,495 | 22,529 | 58,356 | 22,959 |
| 37,316 | 39,453 | 79,451 | 31,253 |
| 34,214 | 38,112 | 217,221 | 39,301 |

**Fig 3G IL-13 in BAL**

| Control | Ex | Bleo | Bleo+Ex |
| --- | --- | --- | --- |
| 75,990 | 130,666 | 157,401 | 103,622 |
| 65,935 | 65,935 | 409,456 | 80,537 |
| 71,145 | 27,239 | 127,951 | 84,834 |
| 75,145 | 125,656 | 152,897 | 81,254 |
| 88,484 | 67,434 | 157,551 | 125,175 |
| 71,890 | 27,051 | 439,421 | 101,542 |
|  |  | 122,551 | 79,537 |
|  |  | 156,037 |  |
